# The role of primary healthcare physicians in violence against Women intervention program in Indonesia

**DOI:** 10.1186/s12875-019-1054-0

**Published:** 2019-12-04

**Authors:** Nuretha Hevy Purwaningtyas, Guswan Wiwaha, Elsa Pudji Setiawati, Insi Farisa Desy Arya

**Affiliations:** 1 Family Medicine Department, Faculty of Medicine, Universitas Brawijaya, Malang, Indonesia; 2 Public Health Department, Faculty of Medicine, Universitas Brawijaya, Malang, Indonesia; 3 Primary Care Medicine Residency Program, Faculty of Medicine, Universitas Padjadjaran, Bandung, Indonesia; 4 Public Health Department, Faculty of Medicine, Universitas Padjadjaran, Bandung, Indonesia

**Keywords:** Puskesmas, Primary healthcare center, Violence against women, Physician role

## Abstract

**Background:**

Violence against women (VAW) has many impacts on health, but the role of the primary healthcare physicians in the intervention program is lacking. This research aimed to explore the primary healthcare physician role in a comprehensive intervention program of VAW in Malang City, Indonesia.

**Methods:**

This qualitative research was conducted using a phenomenology approach. A focused group discussion followed by in-depth interviews were carried out involving six primary healthcare physicians in Puskesmas (Primary Healthcare Center) and two stakeholders. Legal document related to VAW was reviewed to measure up the role of the primary healthcare physicians.

**Result:**

Our study revealed that the role of physicians in primary healthcare centers on the VAW intervention program was limited. This was due to the insufficient knowledge of the physicians on the VAW program, physicians’ constraint on counseling skill, unsupportive infrastructure, and a limited number of physicians in Puskesmas. Some barriers related to the VAW program management were also discovered and needed intervention at the decision-maker level.

**Conclusion:**

The role of primary healthcare physicians in the comprehensive intervention of the VAW program is not optimum. The source of the problem involves the physician capability and program management aspects in all levels of decision-makers. Local government awareness and commitment are needed to improve the overall management of the VAW intervention program in this city.

## Background

Violence against women (VAW) is one of the major public health problems and human rights violations. It forms in many ways, such as intimate partner violence (IPV), IPV during pregnancy, sexual violence (including first force sex), women trafficking, child sexual abuse, and female genital mutilation [1]. World Health Organization (WHO) in its global plan action document mentioned that VAW happens at different stages of woman’s life, including violence by intimate partner and family members, sexual violence by non-partners, trafficking (including sexual and economic exploitation), femicide for various reasons, acid throwing, and sexual harassment in public place, including through social media and online abuse [2].

VAW has many impacts on health, ranging from minor physical damage to major effect, which causes death. Psychological impairment can also be found as VAW impacts, such as post-traumatic stress disorder (PTSD), depression, substance misused, and suicide temptation [3]. Some consequences in the reproductive health aspect were experienced by violence survivors, including unwanted pregnancies, which usually lead to unsafe abortion, sexually transmitted infection (STI), and other gynecological problems [2]. Study conducted by Jalal K. Damra in Jordan revealed that the occurrence of abortion has a significant interaction with psychological violence, and the level of depression of the women is significantly related with the type of violence they received [4], while Karen Devries et al. reported that violence against women is strongly associated with suicide attempts [5].

The health sector is supposed to be one of the entry points for women who survive from violence to seek treatment [2, 6]. The role of the health sector varies, starting from preventing to responding to the case of VAW, such as advocating for a public health perspective, identifying and providing a comprehensive intervention, developing, implementing, monitoring, and evaluating VAW program intervention in health sectors [2]. Research conducted in Sao Paulo, Brazil, found a high utilization of healthcare services by survivors of VAW, particularly for repetitive IPV, which was also confirmed by another study in Brazil [7].

Health sector response needs a strategic direction since it cannot stand alone. WHO in its global plan action to address interpersonal violence stated that there are 4 strategic directions, as follows: [1] strengthening health system leadership and governance, [2] strengthening health services delivery and health workers/providers’ capacity to respond, [3] strengthening the prevention program, and [4] improving information and evidence [2]. Research regarding health sector responses to VAW intervention showed varied results. A research conducted in the UK showed that only less than 50% of health providers (nurses or physicians) gave counseling or education related to VAW, nor referred to specific VAW service providers. It showed a poor situation among health providers in responding to VAW [8]. Supporting the result, a systematic review by O’campo et al. showed that a successful intervention program of VAW needs a comprehensive program approach, significant institutional support, effective screening protocol, thorough initial and ongoing training, and immediate access/referral to onsite and/or offsite support service [9].

Many types of research on physician role in responding to VAW have been conducted overseas, but very little in Indonesia. Hence, this study was trying to explore the physicians’ role in Primary Healthcare Center (Puskesmas) in responding to the violence against women cases.

## Methods

This research was qualitative using a descriptive phenomenology approach [10] to explore physician knowledge, experience, and barrier they faced when handling suspected cases of VAW. Data were obtained through a focus group discussion (FGD) followed by in-depth interviews for deeper exploration of specific findings.

The research was conducted in February 2019 in Malang city, East Java, Indonesia. We used Puskesmas as the entry point since Puskesmas is the main primary healthcare services owned by the government. Puskesmas serves urban community that has various levels of socioeconomic characteristics. Most of the Puskesmas are reachable in terms of the distance. They also manage clinical cases and various programs mandated by the Ministry of Health, including violence against women and children program.

Participants of the research were the physicians who worked in Puskesmas in Malang city, in which one physician represented one Puskesmas. In total, 15 physicians were invited to the focus group discussion. Other stakeholders that were the representations of P2TP2A (Integrated Service Center for Women Empowerment and Children) and Women Crisis Center (an independent organization who work in VAW issues) were also invited to the FGD process. The inclusion criteria for focus group discussion are presented in Table 1.

**Table 1:** The inclusion criteria of the Focus Group Discussion’s Participants

Puskesmas	Other stakeholders
• Physician • Working in Puskesmas in Malang city • Managing clinical cases in Puskesmas	• Working in VAW issues for more than one year • Managing VAW cases in Malang city

An in-depth interview was conducted for one physician and one program manager in Puskesmas who have had managed the violence against women cases for deeper exploration of their experience.

The focus group discussion was attended by five physicians (out of 15 physicians invited) and three representatives from the P2TP2A & women crisis center. The participants’ characteristics are described in Table 2.

**Table 2:** Participants of Focus Group Discussion’s Characteristic

Code	Institution	Sex	Experience in VAW (years)
Focus Group Discussion Participants			
R1	PHC1	Female	> 5 years
R2	PHC2	Female	> 5 years
R3	PHC3	Female	> 5 years
R4	PHC4	Female	> 5 years
R5	PHC5	Female	< 5 years in the recent PHC
CP1	P2TP2A	Female	> 5 years
CP2	P2TP2A	Female	> 5 years
CP3	Women Crisis Center	Female	> 5 years
In-Depth Interview Participants			
DF6	PHC6	Female	> 5 years
PP6	PHC6	Female	> 5 years

R: Resource Person (Physician); CP: Confirming Person; DF: Physician from experienced PHC; PP: Program Coordinator from experienced PHC (midwife)

Data were collected through a focus group discussion and in-depth interviews. Data were recorded and transcribed textually. The data saturation was shown by the homogeny of the participants, and no new information was gained during the session. Transcribed data of a focus group discussion and in-depth interviews were read thoroughly, grouped into categories and sub-categories, and presented descriptively. Data were analyzed manually using Colaizzi’s method of analysis [11]. Peer briefings between 4 researchers were conducted to reach an understanding, and dissent was resolved based on the strongest arguments (empiric and/or evidence based).

This research obtained ethical clearance from Research Ethic Commission Faculty of Medicine Universitas Brawijaya No. 31/EC/KEPK/02/2019.

## Result

There were four categories extracted from the transcription that are the participants’ perception of violence against women, the participants’ recognition of VAW intervention program at their Puskesmas, the physicians’ action when they found suspected VAW case, and the barriers physician faced in managing suspected VAW cases in Puskesmas. The result categories and sub-categories were presented in Table 3.

**Table 3:** Result Categories and Sub-categories extracted from Focus Group Discussion and In-depth Interview

Category: The participants’ perception of Violence Against Women (VAW)	
Sub-Category	Quotes
Correct perception of VAW case	“*In my personal perception, violence against women is not physical only, but perhaps also mentally. For example, in a domestic relationship, when the husband is yelling at his wife, it’s also a part of violence or domestic violence. It could also happen to their child. So, in my personal opinion, it is not only just physical but also psychological violence*”. (R2)
Incorrect perception of VAW case or doubt	“*But if she got violence from the parent in law, is it included* (in VAW)?” (R2) *“… and there was one case, a girl in senior high school, got a stab in her stomach by her school friend... But other than that, I never found any violence against women cases or domestic violence; maybe they are afraid or ashamed to visit Puskesmas (PHC). But in that girl’s case, because it has already liquid flowing from her abdomen, I referred her to the hospital*” (R3)
Category: The participants’ recognition of VAW intervention program at their Puskesmas	
Sub-Category	Quotes
The physician recognized the program	“*Yes, we have the program in our Puskesmas, the PIC for the program is our midwife*” (R1)
The physician did not recognize the program	“*I think the program exists, but we never know because we never have the case…*” (R3)
Category: Physicians’ action when they discover a suspected VAW case	
Sub-Category	Quotes
Providing physical treatment to the patient (of physical violence case)	“*As a doctor, I gave her therapy at that time, and I suggested her to report the case to the police officer*.” (R2)
Reporting to the relating parties, such as teacher, school headmaster, and the police officer	“*I suggested her to report the case to her teacher because I think if the case involved the police officer, it became complicated*.” (R3)
Referring to hospital	“*For the complete examination purposes, I referred the children to the hospital*” (R4) – (Sexual harassment case on a female toddler)
Peaceful settlement	“… *because they didn’t want to proceed to legal suing, the perpetrator is their relatives though*…” (CP2)
Category: Barriers faced by the physicians in managing suspected violence against women cases	
Sub-Category	Quotes
No training available	All participants mentioned that they never received any training related to VAW intervention (altogether) “*We couldn’t call it training, because if it was training, then it should be intensive, but it was almost like only material refreshing*” (PP6)
No Standard Operational Procedure (SOP) available	“*if it is SOP, it seems not existed*” (R1-R5 altogether) “*Actually, I had socialized it to the head of PHC about the intervention pathway… it existed. So, if there’s a violence case, we will know where to go*…” (CP2) “… *we have no official SOP yet, but we have reported it to the sector level for the VAW case management*” (DF6)
Inadequacy of physicians in Puskesmas	*Sometimes it depends…, because there were so many patients in the Puskesmas, and the doctor is only one, so it’s impossible for us to give education to the patients, it takes too long... and when you just alone, you have to handle hundreds of patients, until what time do we have to work? So, that’s why we never explore deeper; we focus more on the main complaint… (R3)*
Lack of infrastructure (no private room)	“*One room for two programs. That situation made the patient unable to tell us the story. We couldn’t even determine whether she is “miss or Mrs.,” they became ashamed because there were so many people in the room, two doctors, nurses, and also male nurses*” (R3) “*We take the patient to a special room; we have HIV counseling room, or Nutrition Counselling room, whatever empty room available for us to be able to interview the patient privately*” (DF6)
*Visum et Repertum* (VeR) or forensic medical examination cannot be conducted in Puskesmas	“*This is one thing that we frequently ask because PHC is actually also able to do that (VeR), for example, physical examination. But, the investigator and the Police Department don’t want that. So, legal or approved visum (forensic medical examination) is the result of the forensic unit in the hospital. Meanwhile, the request is quite a lot, and the visum cost is very expensive*” (CP2) “*Yesss… if in the (Malang) regency PHC can do (VeR), why in the city we can’t do that?*” (CP3)
Attention scarcity among stakeholders	“*We didn’t blame Puskesmas because perhaps District Health Offices rarely conduct evaluations on this program. We will try to remind them through coordination meetings later*…” (CP2)

R1 – R5: Physician who worked in Puskesmas (Primary Healthcare Center); CP1 – CP2 – CP3: Confirming person from other stakeholder; DF: Physician from experienced PHC; PP: Program Coordinator from experienced PHC (midwife)

### Perception of violence against Women case

Some physicians were able to mention that violence against women was not only just physical violence but also in the form of verbal abuse, but even they were able to state the definition correctly, they were in doubt or unable to recognize the real case as VAW.

*“In my personal perception, violence against women is not physical only, but perhaps also mentally. For example, in a domestic relationship, when the husband is yelling at his wife, it’s also a part of violence or domestic violence. It could also happen to their child. So, in my personal opinion, it is not only just physical but also psychological violence”. (R2).*

*“But if she got violence from the parent in law, is it included (in VAW)?” (R2).*

Participant no-3 (R3) shared her story that one day, she managed a case of a schoolgirl who was stabbed by her classmate, and yet she mentioned that it was not a VAW case.

*“… and there’s one case, a girl in senior high school, got a stab in her stomach by her school friend... But other than that, I never find any violence against women cases or domestic violence; maybe they are afraid or ashamed to visit the Puskesmas (PHC). But in that girl’s case, because it has already liquid flowing from her abdomen, I referred her to the hospital” (R3).*

### Recognition of violence against Women intervention program at Puskesmas

There was only one physician who did not know that there was a VAW intervention program at Puskesmas. Apart from being a recognized program, they mentioned that they lacked coordination. The violence against women intervention program in Puskesmas was under the Maternal and Child Health Department, in which the coordinator of the department is the physician.

*“For the maternal and child health program, the coordinator should be the doctor, am I right? So, the (VAW) program manager is supposed to report or coordinate with them”. (CP2).*

### Physician action when discovering suspected VAW cases

There were several possibilities when the physician was managing a case related to violence, namely [1] the physician gave physical treatment to the patient (for physical violence case), [2] the physician suggested the patient report the case to the relating parties, such as teacher/headmaster and the police officer, [3] the physician referred the patient to the hospital for forensic medical examination, [4] the patient refused the physician suggestion and chose for a peaceful settlement. The result from an in-depth interview regarding the case management of VAW covered those 4 points.

*“For reporting purposes, we report the number of VAW cases to the DHO, but for further intervention, we collaborate with P2TP2A (Integrated Service Center for Women Empowerment and Children) where a full team is available” (PP6).*

*“If there is violence, they can also directly report it to the police department. Perhaps they need protection, kept away from family; they can also be referred to the women crisis center. There will also be a psychologist and this.. this.. complete team”. (PP).*

**Fig. 1:**
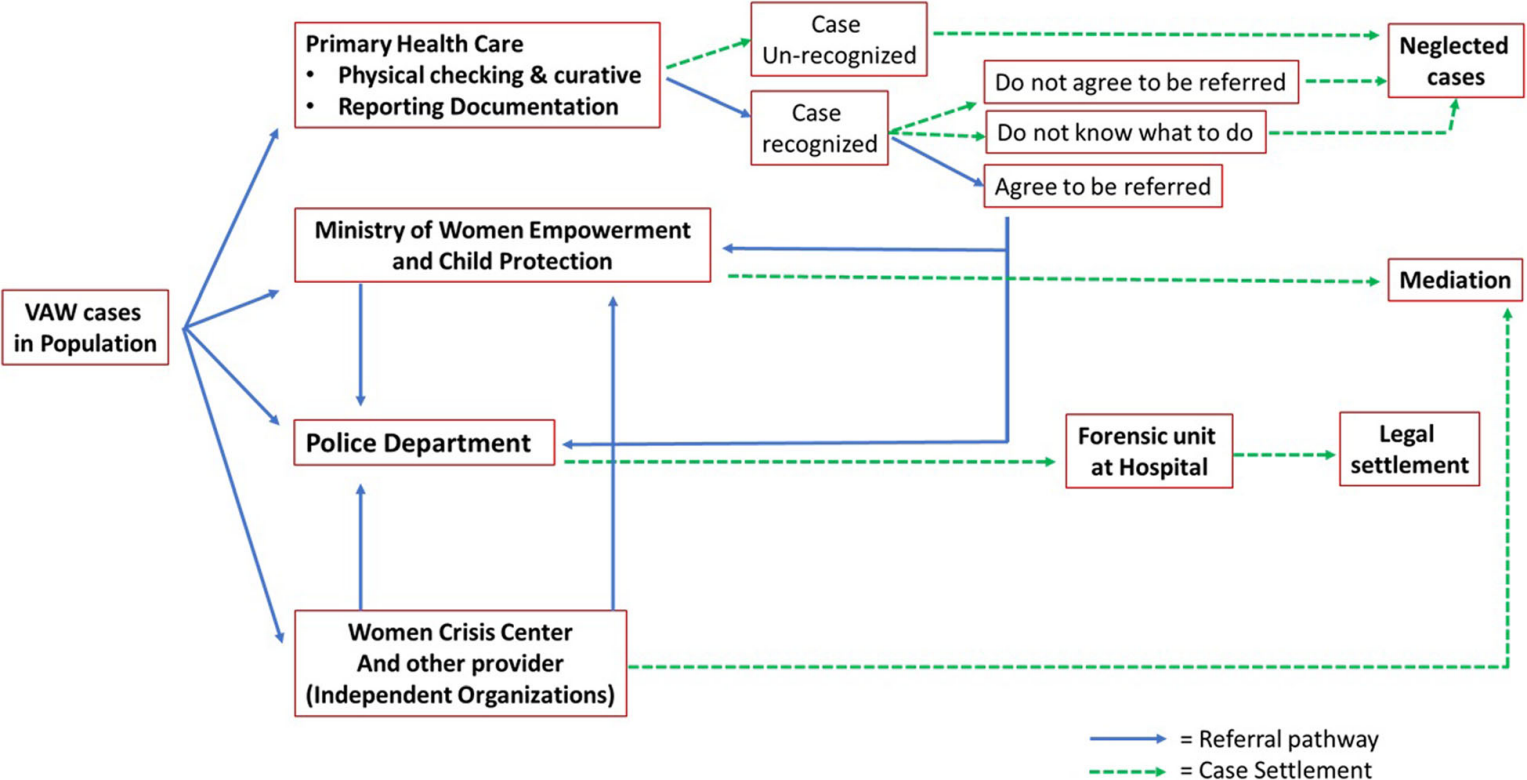
Intervention Pathway of Violence against Women Case in Malang City

Through this result, we were able to develop a factual pathway of VAW case in Puskesmas and community in general as presented in Fig. 1.

### Barriers faced by physicians in managing suspected cases of VAW

This research also explored the barriers experienced by the physician when working on VAW cases. There were six identified problems both in the FGD and the in-depth interview session, explicitly:

#### No training is available

According to the physicians who participated in the FGD, they have never attended training related to VAW case management at the Puskesmas. On the other hand, the confirming person from P2TP2A mentioned that their unit had conducted a regular meeting annually to introduce and raise the awareness of the VAW intervention program to Puskesmas staffs, but she was not sure whether the DHO had a specific training related to it.

*“We never had counseling training before, how to search for deeper information… not yet, and we also don’t know what to do when we get the case, there is no such pathway …”. (R2).*

*“Oh... if it’s socialization, I have conducted it previously, but if it’s training from District Health Office, when I asked the DHO program manager, they said they did it. But perhaps for MCH dept, perhaps the midwives… “. (CP2).*

#### Standard operational procedure (SOP) is not available/not recognized

Physicians in the Puskesmas did not recognize any SOP related to the VAW intervention program. This situation was also strengthened by the physician from Puskesmas that have managed the VAW case. However, the confirming person from P2TP2A said that the SOP existed, and she had shared it with the Puskesmas staffs. Her statement was argued by all physicians.

*I had socialized it (the SOP) to the head of Puskesmas; there was also the intervention pathway… it is (existed), so if we got the case, we know where we have to go, like that.. (CP2).*

*We don’t have official SOP, but at the internal sector meeting, we shared that we have the pathway for the cases management (DF6).*

#### Physician inadequacy in Puskesmas

Intervening violence cases needed a special approach since the issues were considered sensitive, particularly for the victim. Puskesmas serves hundreds of patients every day, hence the physicians said that they do not have enough time to do the anamnesis deeper and to give the patients suggestion regarding their violence case.

*Sometimes it depends…, because there were so many patients in the Puskesmas, and the doctor is only one, so it’s impossible for us to give education to the patient, it takes too long... and when you are just alone, you have to handle hundreds of patients, until what time do we have to work?, so that’s why we never explore deeper, we focus more to the main complaint… (R3).*

#### Lack of infrastructure

Violence case is sensitive, so everyone is not willing to reveal it publicly. The availability of the counseling room is needed, but not every Puskesmas has it. An examination room at the Puskesmas was an open room, which was almost always full by medic and paramedic staffs. This situation prevented the patients from expressing their problems and receiving appropriate supports they needed.

*“One room for two programs, that situation made the patient unable to tell us the story. We couldn’t even determine whether she is “miss or Mrs.,” they became ashamed because there were so many people in the room, two doctors, nurses, and also male nurses” (R3).*

*“We take the patient to a special room; we have HIV counseling room, or Nutrition Counselling room, whatever empty room available for us to be able to interview the patient privately” (DF6).*

#### Visum et Repertum (VeR) or forensic medical examination cannot be conducted in Puskesmas

This issue was brought by the confirming person who compared the situation between Malang regency and Malang city. The forensic medical examination is one of the legal aspects needed when the victims of violence want to proceed with litigation. This procedure affected more to the violence victim, not to the Physician.

#### Attention scarcity

The violence against women intervention program did not get adequate attention. This was shown through the minimal participants who attended the FGD invitation. The FGD was only attended by five physicians (33%) and three people from P2TP2A & Women Crisis Center. The reason stated by one of the physicians who was not attending the FGD mentioned that many papers on her desk buried the invitation. During the discussion, one of the participants also stated that violence against women program was not part of the routine meeting agenda since it was not a priority program.

*“It is not included in the meeting because it’s not a “sexy program,” so it’s neglected a little bit” (CP2).*

## Discussion

The health sector holds an important role in intervening the violence against women cases since the survivor who had physical injury will most likely access healthcare services [12]. Unfortunately, in Indonesia, the health sector pays little attention to VAW intervention programs, and the lack of coordination adds to the catastrophic conditions.

The guideline released by UNFPA and several other UN agencies in 2015 mentioned there were six points that can be provided by essential healthcare services, namely [1] identification of survivors of intimate partner violence, [2] first-line support, [3] care of injuries and urgent medical treatment, [4] sexual assault examination and care, [5] mental health and assessment and care, and [6] documentation. Those services will be able to be provided by any health provider who has been trained and knowledgeable in violence against women issues [13]. Our finding shows that our physician had a limited understanding of violence against women. They were able to mention the basic definition but were unable to identify the factual case. In response to it, they just provided the care of injuries and urgent medical treatment (as no 3 above), while the other services were neglected. One of the impacts in the documentation aspect is that the inability to recognize the VAW case which will affect the reported number of the case will be seen as few. This is an important issue to be highlighted since the few-reported number could be used as an advocacy material for a local government to strengthen the health policy supporting VAW intervention [14].

**Fig. 2:**
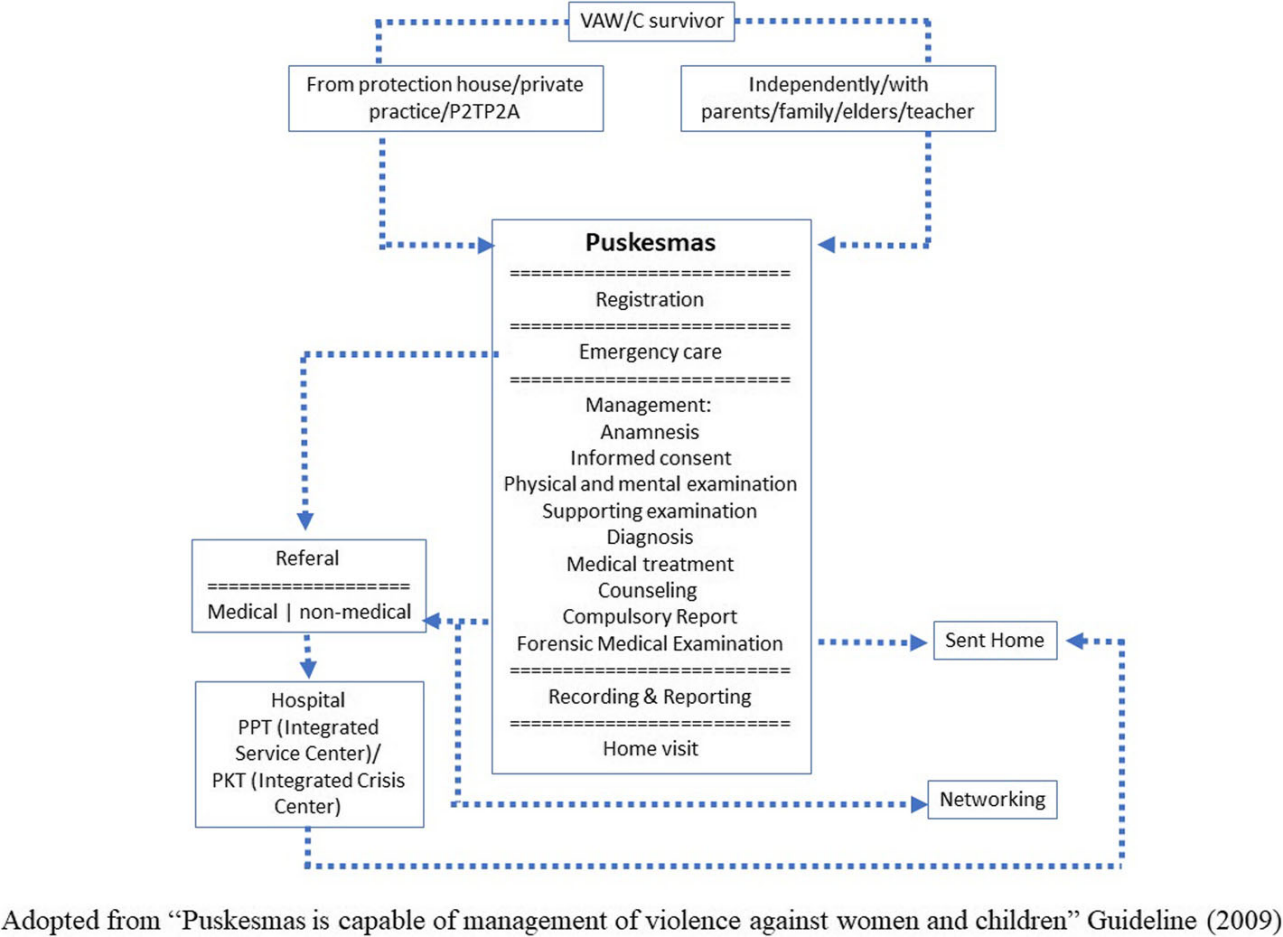
Violence against Women/Children Case Management Algorithm in Puskesmas

One positive finding is that even though the physician has difficulties in recognizing the case, they do know the existence of VAW intervention program in their Puskesmas. The existence of the program showed that the District Health Office response to the Republic of Indonesia Law No. 23 year 2004 concerning the Elimination of Domestic Violence [15]. However, their knowledge of the existing program was very limited, for example, they did not know the standard operational procedure for VAW case management in Puskesmas, while Ministry of Health has had released the management guideline in their development guidance book entitled “Puskesmas is capable of management of violence against women and children” in 2009. In the guidance, we found the algorithm of case management of VAW/Children in Puskesmas as Fig. 2 below: [16].

The possible reason that the physicians in the Puskesmas did not know the standard operating procedure, as mentioned in the FGD, is because of lack of coordination. District Health Office conducted the socialization to the head of Puskesmas or program manager, but the internal coordination within the Puskesmas did not run well. Thus, internal coordination among VAW intervention program managers and other health providers within the institution is crucial. In fact, not only internal coordination is a matter but also external coordination and collaboration among stakeholders [17]. Stakeholders related to violence against women intervention program are regulated by the Presidential Decree No. 18 year 2014 [18] and other local regulations.

Responding to the violence case, most of the physicians mentioned that they treated the physical trauma of the survivor. Some of them also suggested the survivor report the case to the police department or other related institution, although their suggestion was not recorded/reported. This was due to their unawareness of the existing standard operating procedure (SOP). However, the intervention pathway, which was constructed as the result of this research, is almost similar to the existing SOP in the guidance of the Ministry of Health [16].

**Fig. 3:**
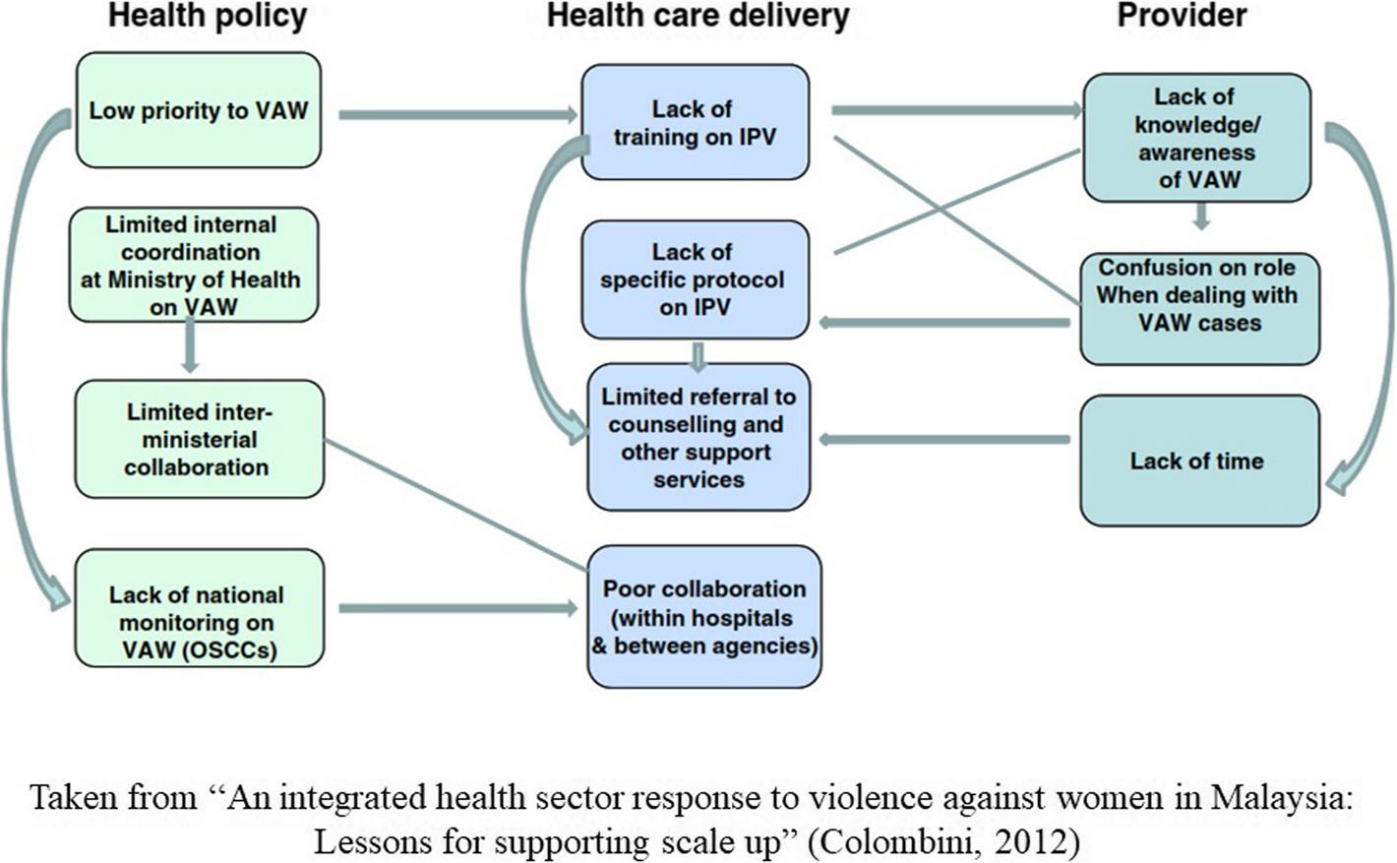
Flowchart on the knock-on effect of different challenges across levels

Our finding on the barriers faced by the physicians in Puskesmas has similarities to the research conducted by Colombini et al. (2012). They mentioned some challenges found in their OSCC (One Stop Crisis Center) model as in Fig. 3 [19].

Lack of physician, infrastructure, and training for the physician are the root cause of inappropriate management of violence against women cases in Malang city, which leads to the domino effect of missing cases. Meanwhile, attention scarcity can be the result of the effect, but also can be the key problems of all neglected factors of VAW intervention program.

Attention scarcity occurred at all levels, starting from health service provider to the central government level. Our finding is strengthened by government statement in the supplement of Decree on Minimum Services Standard for Integrated Services for Women and Children Victims of Violence No. 1 year 2010, which describe that the health services response were substandard because violence against women/children is not a direct health problem [20]. Colombini also found the same result from his research in Sri Lanka (2018) that the health sector did not put the VAW intervention program as a priority. The study found that the network and low engagement of government in the health sector were the important factors that delayed the policy response [21]. A research conducted by Coradi (2016) found that the role of the state is important, and so does the grass-root movement [22]. Therefore, the involvement of the government role in responding to violence against women is crucial.

### Limitation of the research

The small number of participants who attended the focus group discussion from the total invitation showed the lack of health care providers’ attention in the issue of VAW, but this also showed the limitations of this study. The gender homogeneity of the participants was also a constrain, which resulted in an imbalance perspective of VAW program intervention problems because it was dominated by women who mostly are the victims of the violence. Therefore, further research that involves more participants from various stakeholders in Malang City is needed.

## Conclusions

Physician in the primary healthcare center plays an important role in the management of Violence against Women. However, the role is not optimal due to the various challenges at all levels, starting from the healthcare provider, the healthcare institution, the local/central government, and other stakeholders. Local government awareness and commitment are needed to improve the overall management of the Violence against Women intervention program.

## Data Availability

The datasets used and/or analyzed during the current study are available from the corresponding author on reasonable request.

## Abbreviations

BKKBN: Badan Kependudukan dan Keluarga Berencana Nasional (National Family Planning and Population Bureau)
DP3AP2KB: Dinas Pemberdayaan Perempuan, Perlindungan Anak, Pengendalian Penduduk Dan Keluarga Berencana (Women Empowerment, Child Protection, Population Control and Family Planning Office)
FGD: Focus Group Discussion
IPV: Intimate Partner Violence
OSCC: One Stop Crisis Center
P2TP2A: Pusat Pelayanan Terpadu Pemberdayaan Perempuan dan Anak (Integrated Service Center for Women Empowerment and Children)
PHC: Primary Healthcare Center, we used this term to represent Puskesmas
PTSD: Post-Traumatic Stress Disorder
Puskesmas: Pusat Kesehatan Masyarakat. It is a community health center, which in this document we use primary healthcare center (PHC)
SOP: Standard Operational Procedure
STI: Sexually Transmitted Infection
VAW: Violence Against Women
VeR: Visum et Repertum
WCC: Women Crisis Center
WHO: World Health Organization
